# Molecular Identification of *Dendrobium* Species (*Orchidaceae*) Based on the DNA Barcode ITS2 Region and Its Application for Phylogenetic Study

**DOI:** 10.3390/ijms160921975

**Published:** 2015-09-11

**Authors:** Shangguo Feng, Yan Jiang, Shang Wang, Mengying Jiang, Zhe Chen, Qicai Ying, Huizhong Wang

**Affiliations:** 1 Zhejiang Provincial Key Laboratory for Genetic Improvement and Quality Control of Medicinal Plants, Hangzhou Normal University, Hangzhou 310018, China; E-Mails: wangshang0205@126.com (S.W.); jmying99@163.com (M.J.); wulv008@163.com (Z.C.); yingqicai@163.com (Q.Y.); 2 College of Bioscience & Biotechnology, Hunan Agricultural University, Changsha 410128, China; 3 Zhejiang Institute of Chinese Meteria Medica, Hangzhou 310023, China; E-Mail: yanjiang1106@hotmail.com

**Keywords:** *Dendrobium*, ITS2, DNA barcode, species identification, genetic relationship

## Abstract

The over-collection and habitat destruction of natural *Dendrobium* populations for their commercial medicinal value has led to these plants being under severe threat of extinction. In addition, many *Dendrobium* plants are similarly shaped and easily confused during the absence of flowering stages. In the present study, we examined the application of the ITS2 region in barcoding and phylogenetic analyses of *Dendrobium* species (*Orchidaceae*). For barcoding, ITS2 regions of 43 samples in *Dendrobium* were amplified. In combination with sequences from GenBank, the sequences were aligned using Clustal W and genetic distances were computed using MEGA V5.1. The success rate of PCR amplification and sequencing was 100%. There was a significant divergence between the inter- and intra-specific genetic distances of ITS2 regions, while the presence of a barcoding gap was obvious. Based on the BLAST1, nearest distance and TaxonGAP methods, our results showed that the ITS2 regions could successfully identify the species of most *Dendrobium* samples examined; Second, we used ITS2 as a DNA marker to infer phylogenetic relationships of 64 *Dendrobium* species. The results showed that cluster analysis using the ITS2 region mainly supported the relationship between the species of *Dendrobium* established by traditional morphological methods and many previous molecular analyses. To sum up, the ITS2 region can not only be used as an efficient barcode to identify *Dendrobium* species, but also has the potential to contribute to the phylogenetic analysis of the genus *Dendrobium*.

## 1. Introduction

*Dendrobium* Sw., one of the most important genera in the family *Orchidaceae*, comprising more than 1000 species [1,2,3,4], is mainly distributed in tropical and subtropical Asia, and Northern and Eastern Australia [5]. All these species are contained in the Conventions on International Trade in Endangered Species of Fauna and Flora (CITES). There are 74 *Dendrobium* species and two varieties in China, mainly distributed in the southern regions of the Tsinling Mountains [3]. Due to its pharmacological significance for human health, with reported effects including clearing away toxic materials accumulated in human tissues, enhancing the body’s immunity, reducing blood sugar levels, and prolonging life [6,7], *Dendrobium* has been one of the most well-known traditional herbal medicinal plants in China for centuries. For the commercial production of medicinal plants, *Dendrobium* plants are subjected to massive collection for trading in the medicine market in China. Nowadays, due to over-collection and habitat destruction, natural *Dendrobium* populations are under severe threat of extinction.

The accurate identification of *Dendrobium* species is critical to their safe utilization and genetic resource conservation. Traditional methods to identify *Dendrobium* species are based on phenotypic observations [8], while morphological characteristics are often affected by environmental and developmental factors [1,9,10]. Particularly, during the absence of flowering stages, the morphological characteristics of many *Dendrobium* species are extremely similar, rendering their differentiation very difficult and sometimes impossible [1,2,5,11]. Therefore, a simple and accurate identification method for *Dendrobium* plants is indispensable.

DNA barcoding is a new technique using a short and standardized DNA fragment to discriminate among species [12,13,14,15,16,17]. Irrespective of the morphological features of samples and the professional level of users, this technique should consistently identify a species [18]. In addition, some studies have reported that DNA barcoding was also applied for the identification of ancient archaeological samples [19,20] and for intraspecific and population studies [21]. In recent years, DNA barcoding has been generally improved as an efficient tool for species identification and has become a trend and area of recent interest for biology systematics and identification [13,14,22]. In plants, several regions of chloroplast DNA sequences (including *matK*, *rbcL*, *psbA-trnH*, and *atpF-atpH* spacer) and the internal transcribed spacer (ITS) region of the nuclear ribosomal DNA have been advocated as potential plant barcodes [13,23,24]. The internal transcribed spacer 2 (ITS2) is situated between the ribosomal genes 5.8S and 28S and probably has a function in the regulation of the transcription of active ribosomal subunits, as this spacer provides structural elements necessary for correct pre-rRNA processing [25,26,27]. Due to its valuable characteristics, including the availability of conserved regions for designing universal primers, the ease of its amplification, and sufficient variability to distinguish even closely related species, ITS2 was recently proposed as the standard barcode for authentication of medicinal plants [13,14,28,29,30,31,32]. In addition, it has been reported that ITS2 provides taxonomic signatures in systematic evolution [22,33,34] and can be used for rapid taxonomic classification [13,14,35].

The aim of the present study is to test whether ITS2 is a valuable marker for the application of barcoding in *Dendrobium*, and to apply ITS2 as a phylogenetic marker to infer genetic relationships among *Dendrobium* species.

## 2. Results

### 2.1. PCR Amplification, Success Rate and Sequence Characteristics

The amplification and sequence success rate of the ITS2 sequences from sampled specimens of *Dendrobium* species was 100%. The lengths of the ITS2 sequences used for the analyses were in the range of 243–258 bp, with an average of 248 bp. The mean GC content was 52.2%, with a range of 44.8%–64.7%. Therefore, the length and GC content of ITS2 sequences from the collected *Dendrobium* species were relatively variable.

### 2.2. Genetic Divergence within and between Species

When we used six metrics (average inter-specific distance, the minimum inter-specific distance, theta prime, average intra-specific distance, coalescent depth and theta) to estimate genetic divergences, the calculated results showed that ITS2 exhibited significant divergence at the inter-specific level (Table 1) to distinguish between closely related species. At the intra-specific level, relatively lower divergence was observed for all three corresponding metrics.

**Table 1:** Analyses of inter-specific divergence and intra-specific variation of the ITS2 (Internal transcribed spacer 2) sequences in 364 samples of 64 *Dendrobium* species.

Measurement	Kimura 2-Parameter (K2P) Value
All inter-specific distance	0.182 ± 0.036
Theta prime	0.192 ± 0.036
The minimum inter-specific distance	0.185 ± 0.035
All intra-specific distance	0.007 ± 0.004
Theta	0.006 ± 0.003
Coalescent depth	0.014 ± 0.006

### 2.3. Assessment of Barcoding Gap

**Figure 1:**
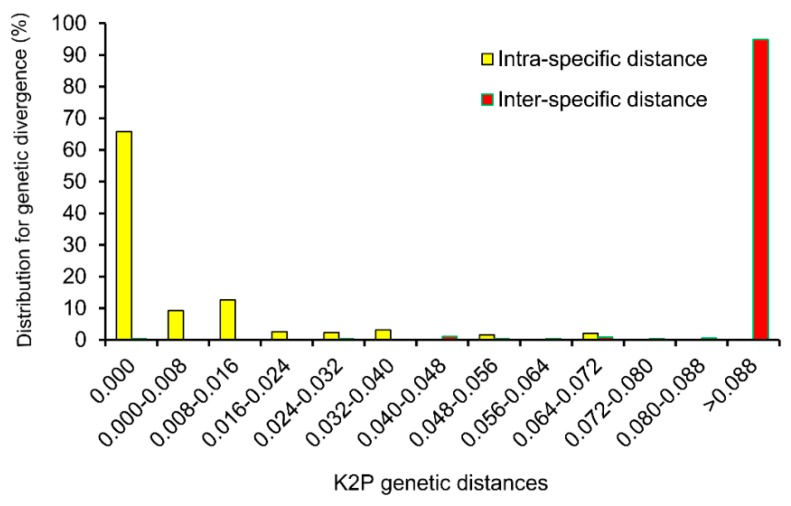
Relative distribution of inter-specific divergence between congeneric *Dendrobium* species and intra-specific variation in the ITS2 region using K2P genetic distance.

To examine intra- *vs.* inter-specific divergence, we investigated the distribution of genetic distance at a scale of 0.008 distance units. Only a slight overlap in inter-/intra-specific variation of ITS2 was found (Figure 1). The inter-specific distance was in the range of 0.000–0.415, equaled zero for only 0.38%, and the proportion of inter-specific genetic distance <0.072 was only 4.18%. The intra-specific distance ranged from 0.000 to 0.072, and most *Dendrobium* species with more than two samples in our study had a unique sequence (65.85%) in the ITS2 region. The results indicated an obvious barcoding gap between inter- and intra-specific divergence, and the ITS2 sequences could provide a useful way to authenticate different *Dendrobium* species.

Wilcoxon two-sample tests also showed significant differences between the inter- and intra-specific divergences (*p* < 0.001). Therefore, ITS2 possessed intra- and inter-specific variation gaps.

### 2.4. Efficacy of ITS2 for Authentication

ITS2 possessed 85.9% and 82.8% identification success rates at the species level for BLAST1 and nearest genetic distance, respectively (Table 2). Thus, ITS2 region exhibited high identification efficiency.

**Table 2:** Comparison of authentication efficiency for ITS2 using different methods.

Methods of Identification	No. of Samples	No. of Species	Correct Identification (%)	Incorrect Identification (%)	Ambiguous Identification (%)
BLAST1	364	64	85.9	0	14.1
Distance	364	64	82.8	0	17.2

### 2.5. Evaluation of the Discriminatory Power of ITS2 Sequences

**Figure 2:**
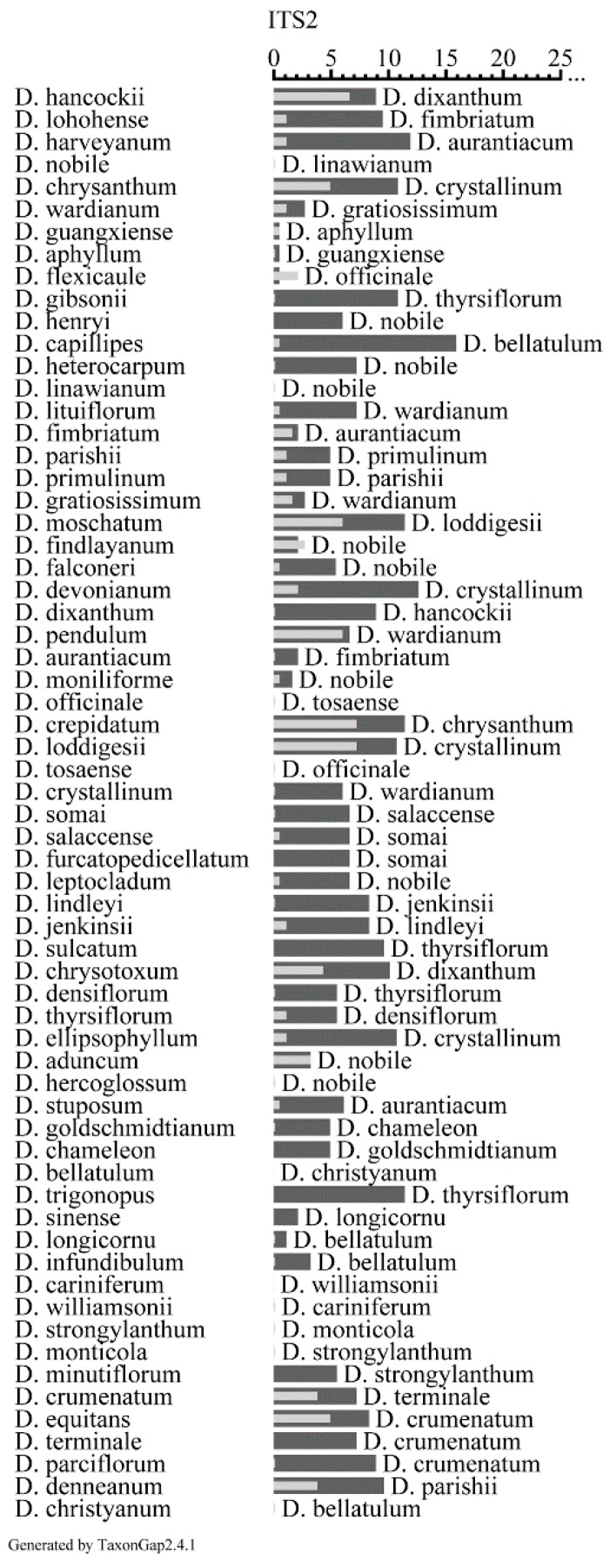
The heterogeneity and separability for individual taxa of ITS2 based on 64 *Dendrobium* species by TaxonGap. The left side shows the complete list of *Dendrobium* species used in this study, including sequences we generated and those retrieved from GenBank (see Table 3 and Table S1). The right side depicts the within species heterogeneity (presented as light gray horizontal bar) and between-species separability (presented as dark grey horizontal bar) values (calculated by using similarity matrix for biomarker ITS2) with different OTUs (Operational Taxonomic Units) as matrix rows for ITS2. The names of the closest relatives (the taxon with the smallest separability) are listed at the right side of the dark grey bar.

The discriminatory power of ITS2 sequences for collected samples was straightforwardly evaluated using software TaxonGap (Figure 2). As a result, over 79.7% of the species had larger inter- than intra-specific diversity; therefore, there were relatively clear species boundaries for ITS2 sequences. However, there were exceptions: 17.2% of the species (see dark grey bar, Figure 2) had identical sequences with their sister-species for *D. nobile vs. D. linawianum*, *D. officinale vs. D. tosaense*, *D. hercoglossum vs. D. nobile*, *D. bellatulum vs. D. christyanum*, *D. cariniferum vs. D. williamsonii*, and *D. strongylanthum vs. D. monticola*.

### 2.6. Phylogenetic Analysis

According to the morphological classification of *Dendrobium* species reported previously [3,5,36], all *Dendrobium* species collected in this study (Table 3 and Table S1) belonged to 12 sections (sects.): *Dendrobium*, *Grastidium*, *Chrysotoxae*, *Distichophyllum*, *Breviflores*, *Stuposa*, *Pedilonum*, *Formosae*, *Stachyobium*, *Crumenata*, *Aporum*, and *Strongyle*. The neighbor-joining (NJ) phylogenetic tree constructed based on ITS2 sequences (Figure S1) grouped all the *Dendrobium* species into four main clusters, of which, however, only two (III, IV) are well supported considering bootstrap values.

**Table 3:** Plant samples of *Dendrobium* used for analysis in this study with voucher number (No.) and GenBank Accession Number.

Species Name	Voucher Number (No.)	Section	Collection Site	GenBank Accession No.
*D. hancockii* Rolfe	D-HZ001	*Dendrobium*	Yunnan	KJ658296
*D. harveyanum* Rchb. f.	D-HZ002	*Dendrobium*	Yunnan	KJ658297
*D. nobile* Lindl.	D-HZ003	*Dendrobium*	Yunnan	KJ658298
*D. chrysanthum* Wall. ex Lindl.	D-HZ004	*Dendrobium*	Guangxi	KJ658299
*D. wardianum* Warner	D-HZ005	*Dendrobium*	Yunnan	KJ658300
*D. guangxiense* S. J. Cheng et C. Z. Tang	D-HZ006	*Dendrobium*	Yunnan	KJ658301
*D. guangxiense* S. J. Cheng et C. Z. Tang	D-HZ007	*Dendrobium*	Guangxi	KJ658302
*D. aphyllum* (Rohb.) C. E. Fishcher	D-HZ008	*Dendrobium*	Guangxi	KJ658303
*D. gibsonii* Lindl.	D-HZ009	*Dendrobium*	Yunnan	KJ658304
*D. gibsonii* Lindl.	D-HZ010	*Dendrobium*	Guangdong	KJ658305
*D. capillipes* Rchb.	D-HZ011	*Dendrobium*	Guangdong	KJ658306
*D. heterocarpum* Lindl.	D-HZ012	*Dendrobium*	Yunnan	KJ658307
*D. fimbriatum* Hook.	D-HZ013	*Dendrobium*	Guangxi	KJ658308
*D. primulinum* Lindl.	D-HZ014	*Dendrobium*	Yunnan	KJ658309
*D. falconeri* Hook.	D-HZ015	*Dendrobium*	Guangdong	KJ658310
*D. devonianum* Paxt.	D-HZ016	*Dendrobium*	Yunnan	KJ658311
*D. dixanthum* Rchb.	D-HZ017	*Dendrobium*	Zhejiang	KJ658312
*D. dixanthum* Rchb.	D-HZ018	*Dendrobium*	Yunnan	KJ658313
*D. pendulum* Roxb.	D-HZ019	*Dendrobium*	Yunnan	KJ658314
*D. moniliforme* (Linn.) Sw.	D-HZ020	*Dendrobium*	Yunnan	KJ658315
*D. officinale* Kimura et Migo	D-HZ021	*Dendrobium*	Yunnan	KJ658316
*D. officinale* Kimura et Migo	D-HZ022	*Dendrobium*	Guangxi	KJ658317
*D. officinale* Kimura et Migo	D-HZ023	*Dendrobium*	Zhejiang	KJ658318
*D. crepidatum* Lindl. ex Paxt.	D-HZ024	*Dendrobium*	Yunnan	KJ658319
*D. loddigesii* Rolfe	D-HZ025	*Dendrobium*	Yunnan	KJ658320
*D. crystallinum* Tchb. F.	D-HZ026	*Dendrobium*	Guangxi	KJ658321
*D. denneanum* Kerr.	D-HZ041	*Dendrobium*	Guangdong	KJ658336
*D. lindleyi* Steudel	D-HZ027	*Chrysotoxae*	Guangdong	KJ658322
*D. lindleyi* Steudel	D-HZ028	*Chrysotoxae*	Yunnan	KJ658323
*D. chrysotoxum* Lindl.	D-HZ029	*Chrysotoxae*	Yunnan	KJ658324
*D. densiflorum* Lindl.	D-HZ030	*Chrysotoxae*	Guangdong	KJ658325
*D. densiflorum* Lindl.	D-HZ031	*Chrysotoxae*	Guangxi	KJ658326
*D. thyrsiflorum* Rchb. f. ex André	D-HZ032	*Chrysotoxae*	Yunnan	KJ658327
*D. aduncum* Wall. ex Lindl.	D-HZ033	*Breviflores*	Gangdong	KJ658328
*D.hercoglossum* Rehb. f.	D-HZ034	*Breviflores*	Guizhou	KJ658329
*D. stuposum* Lindl.	D-HZ035	*Stuposa*	Yunnan	KJ658330
*D. stuposum* Lindl.	D-HZ036	*Stuposa*	Yunnan	KJ658331
*D. longicornu* Lindl.	D-HZ037	*Formosae*	Guangxi	KJ658332
*D. williamsonii* Day et Rchb. F.	D-HZ038	*Formosae*	Guangxi	KJ658333
*D. williamsonii* Day et Rchb. F.	D-HZ039	*Formosae*	Yunnan	KJ658334
*D. christyanum* Rchb. F.	D-HZ042	*Formosae*	Yunnan	KJ658337
*D. christyanum* Rchb. F.	D-HZ043	*Formosae*	Yunnan	KJ658338
*D. strongylanthum* Rchb. F.	D-HZ040	*Stachyobium*	Yunnan	KJ658335

Group I was the most complex, with 42 species, and was further divided into six subgroups. Among which, all species from sect. *Dendrobium* (33 species), *Breviflores* (two species), and *Stuposa* (one species) were included in Group I. Subgroup I-1 comprised 28 species, 25 from sect. *Dendrobium* and the remaining three from sect. *Breviflores* (*D. aduncum* and *D. hercoglossum*) and *Grastidium* (*D. leptocladum*). Subgroup I-2 included one species from sect. *Chrysotoxae* (*D. chrysotoxum*); while I-3 included six species from sect. *Dendrobium* and one from sect. *Stuposa* (*D. stuposum*). Subgroup I-4 contained two species from sect. *Chrysotoxae* (*D. lindleyi* and *D. jenkinsii*). Subgroup I-5 consisted of two species: one from sect. *Dendrobium* (*D. gibsonii*) and one from sect. *Distichophyllum* (*D. ellipsophyllum*). *D. capillipes* from sect. *Dendrobium* and *D. trigonopus* from sect. *Formosae* were assigned into subgroup I-6.

Group II comprised 16 species, and was further divided into four subgroups. Subgroup II-1 contained seven species from sect. *Formosae*. Subgroup II-2 included three species from sect. *Chrysotoxae*. Two species, *D. goldschmidtianum* and *D. chameleon* from sect. *Pedilonum*, constituted subgroup II-3. Compared with subgroup II-1, II-2 and II-3, subgroup II-4 was more complex, and comprised one species (*D. terminale*) from sect. *Aporum*, one species (*D. parciflorum*) from sect. Strongyle, and two species (*D. crumenatum* and *D. equitans*) from sect. *Crumenata*. All species (*D. strongylanthum*, *D. monticola*, and *D. minutiflorum*) from sect. *Stachyobium* constituted a separate group III. The species *D. somai*, *D. salaccense* and *D. furcatopedicellatum* from sect. *Grastidium* were distant from any other *Dendrobium* species, and were assigned into group IV (IV-1).

## 3. Discussion

A rapid and accurate method of species identification is essential to ensure the safe usage of drugs made from medicinal *Dendrobium*, and to preserve *Dendrobium* germplasm resources. To our knowledge, this is the first time that the ITS2 region has been utilized in identification of *Dendrobium* species using such a large sample size.

An ideal DNA barcode should possess high inter-specific but low intra-specific divergence in order to discriminate different species [13,37,38,39]. As in many previous studies [13,14,22,29], we found that ITS2 was a sufficiently variable DNA region among *Dendrobium* species for determination of genetic divergence, and also demonstrated a higher capability of successful discrimination (compared to 85.9% for BLAST1 method and 82.8% for nearest genetic distance method). For example, morphological traits of two species from sect. *Breviflores* (*D. aduncum* and *D. hercoglossum*) were very similar [3], but they could be accurately discriminated based on ITS2 regions. Similar satisfactory results were also obtained for discriminating *D. thyrsiflorum* and *D. densiflorum*, which are difficult to separate using morphological traits [3]. Therefore, we propose that ITS2 can be used for barcoding of *Dendrobium* species. However, it should be noted that some similar morphologic characteristics of *Dendrobium* species might make the classification of these species controversial. Hence, the taxonomic assignment of sequences from GenBank might not be accurate. If these factors were taken into account, the power of ITS2 in species discrimination might be estimated to be lower for *Dendrobium*.

However, ITS2 cannot easily solve all species determination problems in *Dendrobium*. For example, *D. nobile* and *D. linawianum* had identical ITS2 sequences, and another ITS2 sequence was common to *D. officinale* and *D. tosaense*. In addition, the number of *Dendrobium* that cannot be discriminated by ITS2 will probably increase with increasing species or sample set. Thus, other DNA barcodes might be worthwhile as complementary factors for discrimination of these species. 

Recently, *Dendrobium* taxonomy is a global concern of biology systematics and is regarded as one of the most intricate challenges in *Orchidaceae* [4,5,11,40,41]. A number of molecular analyses have indicated that many morphological characters of the *Dendrobium* appear to be homoplasious, and several previously defined infrageneric taxa of *Dendrobium* are not monophyletic [4,5,11,42,43]. In the present study, we found that the ITS2 region was not only useful for DNA barcoding, but could also serve as a valuable standard phylogenetic marker for *Dendrobium* taxonomy. A dendrogram constructed with ITS2 data using the neighbor-joining (NJ) method indicated that the taxonomy of the infrageneric taxa of *Dendrobium* was complicated. As in previous studies [2,5,11,44], we also found that sect. *Dendrobium* was paraphyletic, and also strongly support the view that sects. *Breviflores* and *Stuposa* and *D. chrysotoxum* from sect. *Chrysotoxae* should be included in sect. *Dendrobium* [5]. *D. ellipsophyllum* from sect. *Distichophyllum* was grouped into I-5 together with *D. gibsonii* from sect. *Dendrobium* with middle support (bootstrap support (BS) = 68). Sect. *Distichophyllum* was reported to be a sister group of sect. *Formosae* in a previous study [5]. Since only one species of this section was tested in the present study, more sampling and more evidence is required to determine the relationship between species from sect. *Distichophyllum* and species from other sections. Xiang *et al.* showed that sect. *Chrysotoxae* was probably polyphyletic [5], and we obtained similar results that the collected species of sect. *Chrysotoxae* formed three groups (I-2, I-4, and II-2; Figure S1), and that *D. lindleyi* and *D. jenkinsii*, apart from other species from sect. *Chrysotoxae*, were grouped together (Figure S1, I-4) as Xiang *et al.* reported [5]. Some sections, such as sects. *Stachyobium*, *Pedilonum* and *Formosae* (except *D. trigonopus*), were found to be well supported as monophyletic (BS = 99, 99 and 96, respectively). Three sections, including sects. *Aporum*, *Crumenata* and *Strongyle*, formed the subgroup II-4 with strong support (BS = 97) as previously reported [5,38].

In addition, we found that *D. trigonopus* from sect. *Formosae*, unplaced species reported in previous studies [5,43], were grouped into subgroup I-6 together with *D. capillipes* with weak support (BS < 50). It appears that more sampling and more evidence are required to understand the evolutionary history of *D. trigonopus*. Differing from a previous study [5], we found that sect. *Grastidium* was not well supported as monophyletic. Apart from the other three species of sect. *Grastidium*, *D. leptocladum* was nested with species from sect. *Dendrobium* and grouped into subgroup I-1. Three species, *D. somai*, *D. salaccense*, and *D. furcatopedicellatum* from sect. *Grastidium*, were distant from any other *Dendrobium* species, and were assigned to subgroup IV-1, which may support the view that this section is poorly represented in mainland Asia [5].

Multiple copies of ITS (containing ITS1 and ITS2), one of the main factors that account for incomplete concerted evolution in plants, might cause the question whether the sequence obtained through PCR would be stable and representative, and might result in misleading phylogenetic inferences [14,29,45]. However, we think that the PCR-amplified copies could represent the dominant information of the repeated part of the ITS2 in plant genomes and, ITS2 can be effectively treated as a single locus as previous study [14]. In addition, there are many advantages for its widespread use, such as the levels of variations and multi-copy structure facilitating PCR amplification, even from herbarium specimens [29,46].

## 4. Materials and Methods

### 4.1. Plant Materials

A total of 364 samples of 64 species from genus *Dendrobium* were collected in this study. Among them, 43 specimens of 33 species sampled from the main distribution areas in China were used for sequencing (Table 3), whereas all other data (321 samples) was downloaded from GenBank (Table S1). The collected samples included *D. nobile*, *D. officinale*, *D. fimbratum* and *D. chrysotoxum*, which are listed in the Chinese Pharmacopoeia [6]. The species were verified and confirmed using the specimens stored in the herbarium of the Institute of Botany, Chinese Academy of Sciences, Beijing, China (http://www.nhpe.org). All corresponding voucher samples were deposited in the Zhejiang Provincial Key Laboratory for Genetic Improvement and Quality Control of Medicinal Plants, Hangzhou Normal University.

### 4.2. DNA Extraction, Amplification, and Sequencing

Fresh, young leaves of sampled specimens were randomly collected for genomic DNA isolation. The genomic DNA was isolated as described previously [47]. The ITS2 region was amplified using the following pair of universal primers [29]: ITS-2F, 5′-ATGCGATACTTGGTGTGAAT-3′; and ITS-3R, 5′-GACGCTTCTCCAGACTACAAT-3′. Primers were synthesized by Shanghai Sunny Biotechnology Co., Ltd. (Shanghai, China). PCR was conducted in 25 μL volumes containing 1× PCR Buffer, 2.5 mM Mg^2+^, 0.4 mM dNTPs, 0.5 μM of each primer, 1 U Taq DNA polymerase (TaKaRa Bio., Kyoto, Japan), and 30 ng genomic DNA template. The amplification was performed in a MJ Research PTC-100 thermal cycler (MJ Research, Waltham, MA, USA) with a PCR program: 94 °C for 4 min, followed by 35 cycles of 94 °C for 45 s, 56 °C for 45 s, 72 °C for 1.5 min, and a final extension at 72 °C for 10 min. The PCR products were sequenced by Shanghai Sunny Biotechnology Co., Ltd.

### 4.3. Data Analysis

The original sequences were assembled using CodonCode Aligner V3.0 (CodonCode Co., Centerville, MA, USA). The ITS2 sequences were subjected to Hidden Markov Model (HMM) [48] model analysis to remove the conserved 5.8S and 28S DNA sequences [49]. The sequences with less than 100 bp length and the possible contaminated sequences of fungi were discarded. The ITS2 sequences were aligned using Clustal W [50] and the genetic distances computed using MEGA 5.1 according to the Kimura 2-Parameter (K2P) model [51]. The average inter-specific distance, the minimum inter-specific distance and theta prime were used to represent inter-specific divergences using the K2P model [13,14,52,53]. The average intra-specific distance, coalescent depth and theta were calculated to evaluate the intra-specific variation [13,14,53]. The distributions of intra- *versus* inter-specific variability were compared using the DNA barcoding gaps (The difference between the intra- and inter-specific divergence values is named as “barcoding gap”) [13,14,53]. Wilcoxon two-sample tests were performed as indicated previously [13,23,24]. Two methods of species identification, including BLAST1 and the nearest distance method, were used to evaluate the species authentication efficacy [13,14,54]. In the BLAST1 method, all ITS2 sequences of *Dendrobium* species in this study were used as query sequences. BLAST program (http://blast.ncbi.nlm.nih.gov/Blast.cgi) was used to search for the reference database for each query sequence. Correct identification means that the best BLAST hit of the query sequence is from the expected species; ambiguous identification means that the best BLAST hits for a query sequence were found to be those of several species including the expected species; and incorrect identification means that the best BLAST hit of the query sequence is not from the expected species [14]. In the nearest distance method, correct identification means that the hit in our database based on the smallest genetic distances is from the same species as that of the query; ambiguous identification means that several hits from our database were found to have the same smallest genetic distance to the query sequence; and incorrect identification means that the hit based on the smallest genetic distance is not from the expected species [14]. The discriminatory power of ITS2 sequences in species was calculated using TaxonGAP 2.4.1 software [55].

The phylogenetic analysis for collected *Dendrobium* species based on ITS2 sequences was performed using the neighbor-joining (NJ) method in MEGA 5.1. Bootstrap support (BS) values for individual clades were calculated by running 1000 bootstrap replicates of the data. *Pholidota* and *Bulbophyllum* are closely related to *Dendrobium* in *Orchidaceae* [5,56,57,58]. Four *Pholidota* species (*P. chinensis*, *P. cantonensis*, *P. imbricata* and *P. carnea*) and four *Bulbophyllum* species (*B. orientale*, *B. inconspicuum*, *B. kwangtungense* and *B. omerandrum*) were used as outgroups.

## 5. Conclusions

In summary, our study demonstrated that ITS2 might be a useful DNA barcode to identify *Dendrobium* species, and reconstruct the phylogeny of the genus *Dendrobium*. However, more *Dendrobium* species should be included in the future to verify whether the findings hold when even more closely related taxa are included. Our current work provided much useful genetic information about *Dendrobium* species, which will be useful for germplasm management and resource protection.

## Supplementary Materials

Supplementary materials can be found at http://www.mdpi.com/1422-0067/16/09/21975/s1.
