# Supplementary material for: Molecular Identification of Dendrobium Species (Orchidaceae) Based on the DNA Barcode ITS2 Region and Its Application for Phylogenetic Study
Source: Int J Mol Sci. 2015 Sep 11;16(9):21975–88. doi: 10.3390/ijms160921975 (PMC4613292; doi:10.3390/ijms160921975)
Supplement: Supplementary file 1 [file ijms-16-21975-s001.pdf]

Supplementary Information

Table S1. Accession numbers of the ITS2 sequences of 64 *Dendrobium* species, four *Pholidota* species, and four *Bulbophyllum* species from GenBank.

No.	Sections	Species	Number of Samples	Genebank Accession Numbers
1	*Dendrobium*	*D. hancockii* Rolfe	10	AB593575, HM590377,HQ114259, FJ384726, AF362038, DQ058787, AF362025, JN388591, FJ384725, EU003120
2	*Dendrobium*	*D. lohohense* T. Tang et F. T. Wang	3	AF314135, AF363024, JN388574
3	*Dendrobium*	*D. harveyanum* Rchb. f.	4	HQ114226, AB593576, EU477506, JN388594
4	*Dendrobium*	*D. nobile* Lindl.	13	HQ114219, HQ114217, HQ114218, EF618732, AF362046, AF362028, AF362045, AF362039, AF362037, FJ378649, FJ384727, FJ384728, FJ530948
5	*Dendrobium*	*D. chrysanthum* Lindl.	18	HM054593, HM590376, HM054592, JF713091, HM054597, HM054599, HM054598, HQ114238, AF355572, AF362047, JN388584, JF713093, JF713092, FJ384738, EU003119, HM054594, JF713090, AF314126
6	*Dendrobium*	*D. wardianum* Warner	5	HQ114232, HQ114231, AF420245, JN388600, DQ058789
7	*Dendrobium*	*D. guangxiense* S. J. Cheng et C. Z. Tang	1	GU339108
8	*Dendrobium*	*D. aphyllum* (Roxb.) C. E. Fischer	20	HM054560, HM054558, HM054556, HM054554, HM054552, HM054550, HM054561, HM054559, HM054557, HM054553, HM054551, HM054549, JN388571, HQ114247, HQ114248, AB593539, HM590384, FJ428219, EU840691, AF355573
9	*Dendrobium*	*D. flexicaule* Z. H. Tsi, S. C. Sun et L. C. Xu	2	AF355570, FJ384743
10	*Dendrobium*	*D. gibsonii* Lindl.	1	GU339105
11	*Dendrobium*	*D. henryi* Schltr.	1	EF629323
12	*Dendrobium*	*D. capillipes* Rchb. f.	3	HQ114224, JN388582, AF362035
13	*Dendrobium*	*D. heterocarpum* Lindl.	6	JF713106, JF713105, HM054667, JN388592, JN388593, GU339101
14	*Dendrobium*	*D. linawianum* Rchb. f.	6	HM590371, AB593599, AF521613, EU003117, EU003115, JN388573
15	*Dendrobium*	*D. lituiflorum* Lindl.	2	AB593602, AF355571
16	*Dendrobium*	*D. fimbriatum* Hook	8	EU003116, AF314130, HM054635, HM054632, HM054636, HM054633, HM054637, HM590392
17	*Dendrobium*	*D. parishii* Rchb. f.	5	HM590378, AB593630, HM054735, HM054736, EU121417
18	*Dendrobium*	*D. primulinum* Lindl.	16	HM054756, HM054754, HM054752, HM054750, HM054748, HM054757, HM054755, HM054753, HM054751, HM054749, HM054747, JN388598, JN388597, HQ114242, AB593641, AF362913

Table S1. *Cont.*

No.	Sections	Species	Number of Samples	Genebank Accession Numbers
19	*Dendrobium*	*D. gratiosissimum* Rchb. f.	4	JN388590, FJ384737, DQ058790, AF311780
20	*Dendrobium*	*D. moschatum* (Buch. –Ham.) Sw.	11	HM054716, HM054714, HM054712, HM054710, HM054715, HM054711, HM054709, EF629326, AY485695, AY239983, AF314137
21	*Dendrobium*	*D. findleyanum* Par. Et Rchb. f.	4	JN388589, HQ114257, AF362031, EU477504
22	*Dendrobium*	*D. falconeri* Hook.	3	JF713102, HQ114239, FJ384734
23	*Dendrobium*	*D. devonianum* Paxt.	7	JF713100, JF713098, JF713099, HQ114244, EU477502, FJ384735, AF311779
24	*Dendrobium*	*D. dixanthum* Rchb. f.	2	GU339103, DQ058788
25	*Dendrobium*	*D. pendulum* Roxb.	5	HQ114234, DQ058791, EU477508, AF362912, GU339115
26	*Dendrobium*	*D. aurantiacum* Rchb. f.	2	AF362044, AF362042,
27	*Dendrobium*	*D. moniliforme* (L.) Sw.	4	HQ114246, GU339111, EU003114, AF314136
28	*Dendrobium*	*D. officinale* Kimura *et* Migo	18	HQ114245, GU339109, EU592018, FJ384723, FJ384724, FJ588873, FJ588871, FJ530946, FJ530944, EF221849, FJ588872, FJ530947, FJ530945, EF221854, EF221850, EF221848, AF311776, AF314139
29	*Dendrobium*	*D. crepidatum* Lindl. ex Paxt.	24	HM054624, HM054620, HM054618, HM054614, HM054612, HM054610, HM054608, HM054606, HM054604, HM054623, HM054621, HM054619, HM054617, HM054613, HM054611, HM054609, HM054607, HM054605, HM054603, JN388586, HQ114240, AY842035, AF314128, AF355574
30	*Dendrobium*	*D. loddigesii* Rolfe	9	JN388569, HQ114220, AB593604, EU592016, HM590374, AY485703, EU121418, AF314134, AF311778
31	*Dendrobium*	*D. tosaense* Makino	3	EU003113, HM590367, AF521617
32	*Dendrobium*	*D. crystallinum* Tchb. F.	3	HQ114243, KF143447, GU339116
33	*Dendrobium*	*D. denneanum* Kerr.	9	FJ384731, AF362043, FJ384732, FJ530949, FJ38473, EU840702, AF362040, JN388572, FJ384729
34	*Grastidium*	*D. somai* Hayata	3	HM590380, EU840692, AF521616
35	*Grastidium*	*D. salaccense* (Bl.) Lindl.	2	HQ114260, JN388577
36	*Grastidium*	*D. furcatopedicellatum* Hayata	1	AF521611
37	*Grastidium*	*D. leptocladum* Hayata	3	HM590373, AF521612, EU840697
38	*Chrysotoxae*	*D. lindleyi* Stendel	4	JN388568, GU339114, DQ058784, AF314133
39	*Chrysotoxae*	*D. jenkinsii* Lindl.	8	JF713108, HM054670, JF713109, HM054671, JN388595, HQ114251, DQ058785, AF314132
40	*Chrysotoxae*	*D. sulcatum* Lindl.	1	EU477510

Table S1. *Cont.*

No.	Sections	Species	Number of Samples	Genebank Accession Numbers
41	*Chrysotoxae*	*D. chrysotoxum* Lindl.	10	HQ114221, HQ114223, AF314127, HM590383, HQ114222, AF362023, FJ384736, HM054601, EU477501, JN388585
42	*Chrysotoxae*	*D. densiflorum* Lindl.	3	HM054626, JF713097, AF314129
43	*Chrysotoxae*	*D. thyrsiflorum* Rchb. f.	6	AY240001, HM054760, HQ114227, FJ384733, AF362032, HM054758
44	*Distichophyllum*	*D. ellipsophyllum* T. Tang et F. T. Wang	2	AF362033, AY239965
45	*Breviflores*	*D. aduncum* Lindl.	4	AF314125, GU339110, JN388580, JF713083
46	*Breviflores*	*D. hercoglossum* Rchb. f.	3	AB593580, AF314131, JN388576
47	*Stuposa*	*D. stuposum* Lindl.	3	JN388599, HQ114237, GU339104
48	*Pedilonum*	*D. goldschmidtianum*	2	AY239970, EU840695
49	*Pedilonum*	*D. chameleon* Ames	1	AF521607
50	*Formosae*	*D. bellatulum* Rolfe	1	EU592015
51	*Formosae*	*D. trigonopus* Rchb. f.	1	HQ114228
52	*Formosae*	*D. sinense* T. Tang et F. T. Wang	1	JN388578
53	*Formosae*	*D. longicornu* Lindl.	2	GU339112, DQ058796
54	*Formosae*	*D. infundibulum* Lindl.	2	HM054669, HM054668
55	*Formosae*	*D. cariniferum* Rchb. f.	1	AF362027
56	*Formosae*	*D. williamsonii* Day et Rchb. f.	3	HQ114225, AF314140, AF362030
57	*Formosae*	*D. christyanum* Rchb. F.	2	GU339106, EF629325
58	*Stachyobium*	*D. strongylanthum* Rchb. f.	3	DQ058797, GU339107, FJ384739
59	*Stachyobium*	*D. monticola* P. F. Hunt et Summerh.	2	DQ058798, DQ058799
60	*Stachyobium*	*D. minutiflorum* S. C. Chen et Z. H. Tsi	1	DQ058800
61	*Crumenata*	*D. crumenatum* Sw.	8	HM590370, HM054625, JN388587, AF521608, JF713096, JF713095, AY239963, EU840700
62	*Crumenata*	*D. equitans* Kraenzl.	3	HM590388, EU840701, AF521609
63	*Aporum*	*D. terminale* Par. et Rchb. f.	1	DQ058801
64	*Strongyle*	*D. parciflorum* Rolfe	2	HQ114252, JN388575
65	Outgroup	*P. chinensis* Lindl.	1	EU592035
66	Outgroup	*P. cantonensis* Rolfe	1	AF314141
67	Outgroup	*P. imbricata* Hook	1	AF302737
68	Outgroup	*P. carnea* Lindl.	1	AF302738
69	Outgroup	*B. orientale* Seidenf.	1	JN619416
70	Outgroup	*B. inconspicuum* Maxim	1	AB786895
71	Outgroup	*B. kwangtungense* Schltr.	1	JN619414
72	Outgroup	*B. omerandrum* Hayata	1	JN619419

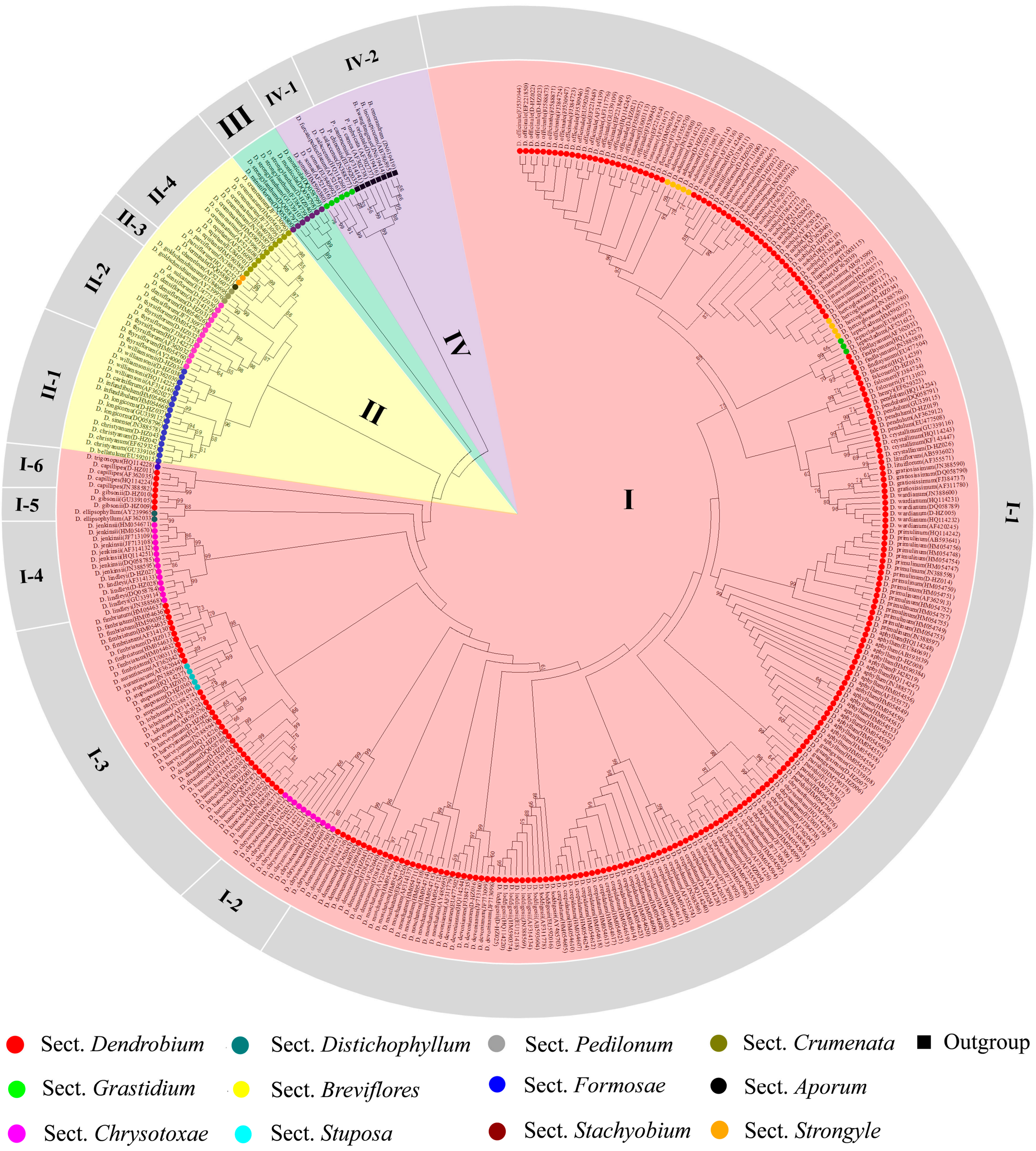

Figure S1. Neighbor-joining (NJ) tree based on ITS2 sequences for *Dendrobium* species. Numbers above branches indicate bootstrap support (BS ≥ 50) values
